# Preservation of whole antibodies within ancient teeth

**DOI:** 10.1016/j.isci.2023.107575

**Published:** 2023-08-09

**Authors:** Barry Shaw, Thomas McDonnell, Elizabeth Radley, Brian Thomas, Lynn Smith, Carol A.L. Davenport, Silvia Gonzalez, Anisur Rahman, Rob Layfield

**Affiliations:** 1 School of Life Sciences, University of Nottingham Medical School, Nottingham, UK; 2 Centre for Rheumatology Research, Division of Medicine, UCL, London, UK; 3 Department of Biochemistry, University of Toronto, Toronto, Canada; 4 Mass Spectrometry Research Group, University of Liverpool, Liverpool, UK; 5 Norton Priory Museum and Gardens, Runcorn, UK; 6 Research Centre for Evolutionary Anthropology and Paleoecology, Liverpool John Moores University, Liverpool, UK

**Keywords:** Biological sciences, Paleobiology, Paleobiochemistry

## Abstract

Archaeological remains can preserve some proteins into deep time, offering remarkable opportunities for probing past events in human history. Recovering functional proteins from skeletal tissues could uncover a molecular memory related to the life-history of the associated remains. We demonstrate affinity purification of whole antibody molecules from medieval human teeth, dating to the 13^th^–15^th^ centuries, from skeletons with different putative pathologies. Purified antibodies are intact retaining disulphide-linkages, are amenable to primary sequences analysis, and demonstrate apparent immunoreactivity against contemporary EBV antigen on western blot. Our observations highlight the potential of ancient antibodies to provide insights into the long-term association between host immune factors and ancient microbes, and more broadly retain a molecular memory related to the natural history of human health and immunity.

## Highlights

• Whole disulfide-linked antibodies can be affinity purified from medieval human teeth
• Purified antibodies retain residual immunoreactivity against contemporary antigens

## Graphical abstract

**Figure:**
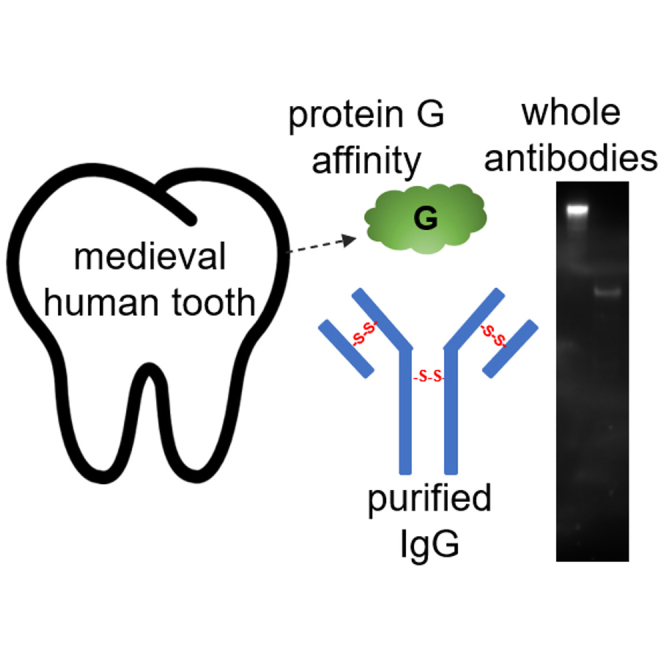

## Introduction

The developing field of palaeoproteomics—the analysis of ancient proteins—recognizes that some proteins are remarkably stable, surviving in, and identifiable from mineralized tissues after millions of years.^1^ Like its subject matter the field has laid dormant until relatively recently, but significant breakthroughs have brought palaeoproteomics to the fore of molecular research, particularly in the areas of species identification and evolutionary relationships.^2^^,^^3^ There are enormous opportunities emerging for ancient protein sequences to provide new insights into species migration, responses to changing environments, reconstruction of ancient diets and lifestyles, as well as human disease across a historic time course.^1^

Ancient proteins present several advantages over the better studied ancient DNA (aDNA). Ancient proteins exhibit increased stability in microenvironments where aDNA may be degraded. Further, the possibility of sample contamination is reduced as ancient protein detection by mass spectrometry (MS) does not rely on any amplification step. Most interestingly, ancient protein analysis permits detection of post-translational modifications (for example phosphorylation^3^) and potentially retention of biological function, offering unrivaled opportunities for exploring the history and evolution of disease.

Typically, palaeoproteomic studies of calcified tissues (bones and teeth) have focused on the most abundant proteins such as collagen, permitting the identification of a ∼160,000-yo Denisovan hominid in China.^2^ Here, a single amino acid collagen variant was able to distinguish the skeletal remains from modern humans and Neanderthals. Non-collagen protein sequences have also been recovered from calcified archaeological samples, including peptide sequences from over 100 different proteins identified from a 43,000-yo mammoth femur.^4^ Palaeoproteomics can reach back into deep time with proteins successfully recovered and sequenced after preservation in >6.5-million-yo ostrich eggshell^5^ and 1.7-million-yo dental enamel from an ancient rhinoceros.^3^ Previously we applied MS-based sequencing to non-collagenous proteins recovered from 800-yo human bones and teeth to permit retrospective diagnosis of a phenotypically unusual form of the skeletal disorder Paget’s disease. In this case we used targeted proteomic analyses to detect > 60% of the primary sequence of preserved ancient human SQSTM1/p62, a contemporary Paget’s disease protein biomarker, with western blotting also indicating protein abnormalities and/or modification.^6^

Studying ancient immunity can provide insights and an evolutionary perspective into immune responses to pathogens and how these have changed over time, and potentially how organisms may respond to new pathogens in the future. To date, although several palaeoproteomic analyses have noted the presence of ancient human antibody peptides from bone (Kendall et al.^7^ and references therein), a detailed assessment of their sequences and function has not been performed. Despite initial optimism that calcified tissues can preserve ancient antibodies with immune reactivity (see discussion), a recent detailed analysis evaluated various methods in parallel for extraction of ancient human IgG protein, concluding poor recovery, evidence of degradation, and overall unsuitability as targets for detecting historical antigens.^7^ However, “degradation” was indicated based on non-tryptic peptide cleavage and deamination of Glu/Asn residues in the antibody sequences, and only through denaturing SDS PAGE analyses, without functional testing of immune reactivity. In short, our work is motivated by the recognition that studies to date have not robustly explored preservation and other elements of the molecular memory of ancient antibodies.

Here, we present data supporting the presence of whole, intact, disulphide-linked antibodies, amenable to affinity purification from medieval human teeth. Moreover, we find purified human ancient antibodies retain apparent residual immunoreactivity against contemporary antigens on western blot. Antibodies can also be recovered from older non-human bone, raising the possibility that functional antibody preservation may be a common, overlooked feature of calcified archaeological skeletal samples.

## Results

### Ancient antibodies can be purified from medieval human teeth

Our previous proteomic analysis of medieval human remains from the Norton Priory collection with atypical Paget’s disease, confirmed that bones and teeth are both a rich source of preserved ancient proteins, particularly in the insoluble pellet fractions that remain after sequential extractions of the decalcified matrix.^6^ Here, we extended these proteomic analyses to human teeth from the Chester Greyfriars excavation at Linenhall^8^ from skeletons dating between 1285-1470AD with evidence of putative pathologies including Paget’s disease, rheumatoid arthritis (referred to here as RA-tooth or RA-skeleton), and osteoporosis (Table 1).

**Table 1:** Medieval human teeth analyzed and putative pathologies of skeletal remains

Skeleton	Tooth sampled	C-14 dating	Principal putative pathology
LP2045-002	Right upper second molar	1290-1410AD	Rheumatoid arthritis
LP2045-004	Left upper second molar	1425-1479AD	Paget’s disease
LP2045-018	Right upper second molar	1285-1330/1340-1395AD	Osteoporosis

**Figure 1:**
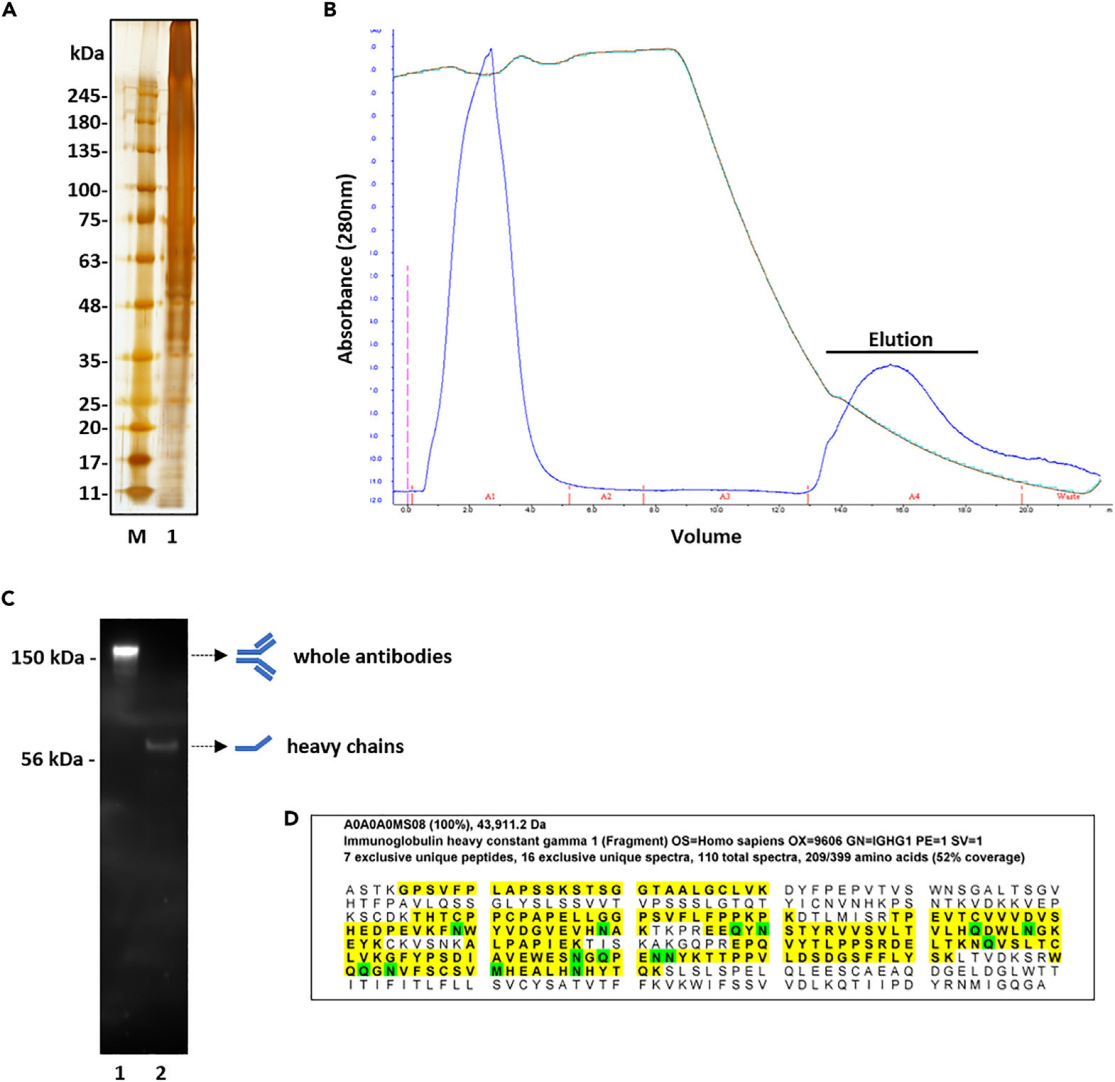
Purification of whole ancient antibodies from human RA-tooth (A) Representative SDS PAGE analysis (silver stain) of proteins extracted from medieval human RA-tooth (M, molecular weight marker; 1, phosphate extraction). (B) FPLC trace of protein G affinity purification from the RA-tooth phosphate extraction, with elution profile showing clear peak (A_280_) corresponding to purified proteins (Elution). (C) Western blot under non-reducing (lane 1) or reducing (lane 2) conditions of affinity purified ancient antibodies from the RA-tooth, with different antibody structures noted. (D) Primary sequence coverage (yellow) of immunoglobulin heavy constant gamma 1 (fragment), IGHG1, from purified antibodies from the RA-tooth, with evidence of amino acid residue deamidation consistent with protein aging (Q and N, green; M, green indicates methionine oxidation).

Complex protein mixtures, as indicated by reducing SDS PAGE, were readily extracted from all decalcified medieval human teeth and were consistently visible (by silver-stain) in the final phosphate-soluble fractions (Figures 1A and S1A). Extracted protein mixtures were amenable to liquid chromatography tandem MS (LC-MS/MS) protein sequencing following tryptic digestion. Proteomic profiling identified numerous proteins across samples, consistently detecting low level human antibody (IgG) peptide sequences in the phosphate-soluble fractions (data not shown), in accordance with other studies (see discussion).

To investigate possible recovery of preserved ancient antibodies, rather than simple shotgun peptide sequence detection, we attempted protein G-based affinity purification of phosphate buffer-soluble proteins extracted from the teeth. Protein G binding of antibodies involves recognition of the Fc portions, and as such successful affinity purification is itself indicative of intact Fc regions. Purified proteins were consistently obtained from phosphate-soluble extracts washed over protein G columns, and eluted peaks (A_280_) were collected for analysis (Figure 1B, data from the putative RA-tooth is shown, representative of other samples).

### Purified ancient antibodies retain disulphide linkages

Abundant intact ancient antibodies were detected in purified fractions under non-reducing conditions using western blotting with anti-human IgG heavy chain antibody as primary, at apparent molecular weights of ∼150 kDa (Figures 1C, S1B, and S1C). These appeared to be whole antibodies, as they co-migrated with contemporary control human IgG-reactive bands (Figure S1B). Whole antibodies were recovered from all three teeth. Western blotting (again anti-human IgG heavy chain as primary) of purified antibodies, now under reducing conditions, revealed immunoreactive bands with an estimated molecular weight of ∼60 kDa (Figure 1C, lane 2; Figure S1C, lane 2). The change of band migration between non-reducing and reducing conditions is entirely consistent with the reduction of disulphide-linked heavy and light chains in the purified whole antibodies. Overall, this indicated the antibodies were preserved intact, retained a degree of structure, and could potentially retain binding function.

We also performed an exploratory analysis of antibody preservation over longer time periods in other skeletal tissues, using bone from a permafrost sample of mammoth (*Mammuthus primigenius*) femur, with a radiocarbon age of ∼37,000 years.^9^ Using elephant serum as a control to optimize methods, protein A purification was found to be superior to protein G, and extracted whole antibodies migrating on non-reducing SDS PAGE at ∼150 kDa (see Figure S2A). Purification of mammoth bone extracts using protein A-based affinity chromatography also revealed purified antibodies migrating on non-reducing western blot at ∼150 kDa (see Figure S2B), consistent with the presence of preserved whole antibodies into deep time in calcified tissues other than teeth.

### Purified ancient antibodies are amenable to protein sequence analysis

We selected purified human ancient antibodies from the putative RA-tooth for a deeper proteomic analysis. Reducing SDS PAGE followed by targeted LC-MS/MS analysis of separate gel slices corresponding to heavy and light chain fractions, identified a total of 206 human proteins in 152 clusters across the two samples (minimum of 2 peptides). Of these proteins, 27 were recognized by the search term “immunoglobulin” (Table 2) with several co-purifying blood proteins also detected (not shown). Primary sequence coverage of individual immunoglobulins was high (as an exemplar, 52% for immunoglobulin heavy constant gamma 1 [fragment], IGHG1, Figure 1D), with evidence of considerable deamidation consistent with protein aging. Antibody sequences extended across a broad range of antibody classes (Table 2). Whether these preserved antibodies represent immune responses to local conditions within the oral cavity and gums, and/or a broader sample of circulating antibodies, is currently unclear.

**Table 2:** Detection of purified ancient human tooth antibody sequences from the RA-tooth (LP-2045-002) extends across a broad range of antibody classes

Protein Name	Accession Number	aCoverage %	bUnique peptides
Immunoglobulin heavy constant gamma 1 (Fragment) OS = Homo sapiens GN = IGHG1 PE = 1 SV = 1	A0A0A0MS08	52	7
Immunoglobulin heavy constant gamma 2 (Fragment) OS = Homo sapiens GN = IGHG2 PE = 1 SV = 1	A0A286YEY4	51	7
Immunoglobulin heavy constant gamma 3 (Fragment) OS = Homo sapiens GN = IGHG3 PE = 1 SV = 1	A0A286YES1	39	6
Immunoglobulin kappa constant OS = Homo sapiens GN = IGKC PE = 1 SV = 2	P01834	80	7
Immunoglobulin heavy constant gamma 4 (Fragment) OS = Homo sapiens GN = IGHG4 PE = 1 SV = 1	A0A286YFJ8	54	7
Cluster of Immunoglobulin heavy constant alpha 1 (Fragment) OS = Homo sapiens GN = IGHA1 PE = 1 SV = 1	A0A286YEY1 [3]	45	11
Cluster of Immunoglobulin lambda constant 2 OS = Homo sapiens GN = IGLC2 PE = 1 SV = 1	P0DOY2 [4]	82	2
Cluster of Immunoglobulin heavy variable 3/OR16-9 (non-functional) (Fragment) OS = Homo sapiens GN = IGHV3OR16-9 PE = 1 SV = 1	A0A0B4J2B5 [7]	42	2
Immunoglobulin heavy constant mu (Fragment) OS = Homo sapiens GN = IGHM PE = 1 SV = 1	A0A1B0GUU9	28	10
Polymeric immunoglobulin receptor OS = Homo sapiens GN = PIGR PE = 1 SV = 4	P01833	6	4
Cluster of Immunoglobulin kappa variable 3–20 OS = Homo sapiens GN = IGKV3-20 PE = 1 SV = 2	P01619 [2]	22	1
Immunoglobulin heavy variable 3/OR16-12 (non-functional) (Fragment) OS = Homo sapiens GN = IGHV3OR16-12 PE = 1 SV = 1	A0A075B7B8	19	1
Cluster of Immunoglobulin heavy variable 4–61 OS = Homo sapiens GN = IGHV4-61 PE = 3 SV = 1	A0A0C4DH41 [8]	21	1
Immunoglobulin kappa variable 3–7 (non-functional) (Fragment) OS = Homo sapiens GN = IGKV3-7 PE = 1 SV = 1	A0A075B6H7	23	2
Immunoglobulin kappa variable 4-1 OS = Homo sapiens GN = IGKV4-1 PE = 1 SV = 1	P06312	30	3
Immunoglobulin lambda variable 3–9 OS = Homo sapiens GN = IGLV3-9 PE = 3 SV = 1	A0A075B6K5	23	3
Immunoglobulin heavy variable 3–49 OS = Homo sapiens GN = IGHV3-49 PE = 3 SV = 1	A0A0A0MS15	28	3
Immunoglobulin J chain (Fragment) OS = Homo sapiens GN = JCHAIN PE = 1 SV = 8	D6RD17	19	3
Immunoglobulin kappa variable 2D-29 OS = Homo sapiens GN = IGKV2D-29 PE = 3 SV = 1	A0A075B6S2	20	2
Immunoglobulin heavy variable 3–15 OS = Homo sapiens GN = IGHV3-15 PE = 3 SV = 1	A0A0B4J1V0	20	2
Immunoglobulin heavy variable 3–72 OS = Homo sapiens GN = IGHV3-72 PE = 3 SV = 1	A0A0B4J1Y9	18	2
Immunoglobulin heavy variable 1–18 OS = Homo sapiens GN = IGHV1-18 PE = 3 SV = 1	A0A0C4DH31	24	2
Cluster of Immunoglobulin lambda variable 3–25 OS = Homo sapiens GN = IGLV3-25 PE = 1 SV = 2	P01717 [2]	19	1
Immunoglobulin heavy variable 1/OR15-1 (non-functional) (Fragment) OS = Homo sapiens GN = IGHV1OR15-1 PE = 1 SV = 1	A0A075B7D0	21	2
Immunoglobulin lambda variable 2–11 OS = Homo sapiens GN = IGLV2-11 PE = 1 SV = 2	P01706	13	2
Immunoglobulin heavy variable 1–3 OS = Homo sapiens GN = IGHV1-3 PE = 3 SV = 1	A0A0C4DH29	22	1
Immunoglobulin heavy variable 1–46 OS = Homo sapiens GN = IGHV1-46 PE = 1 SV = 2	P01743	22	1

a Primary sequence coverage for the particular protein.

b Exclusive unique peptide counts. Combined data from gel slices corresponding to both heavy and light chain fractions. Peptide sequence data for protein IGHG1 is presented in Figure 1D.

### Purified ancient antibodies retain immune reactivity

**Figure 2:**
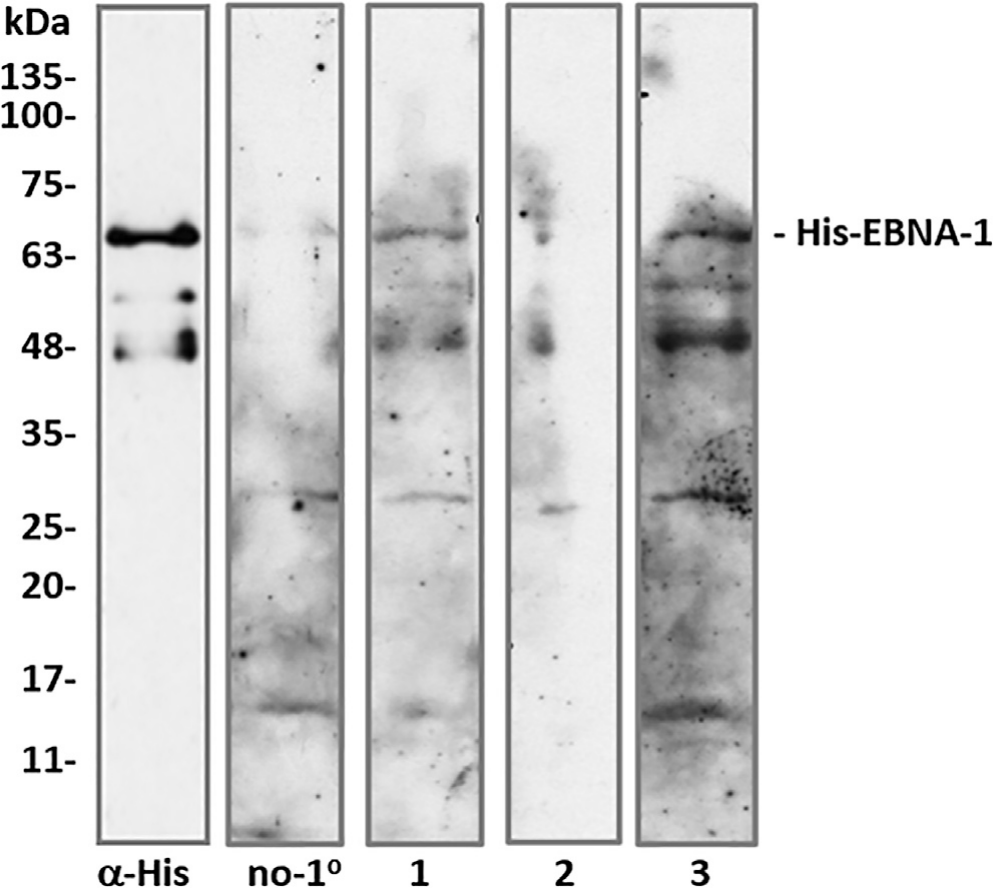
Purified ancient human antibodies retain immunoreactivity on Western blot against recombinant human His-EBNA-1 protein antigen Lane 1, antibodies from RA-tooth; lane 2, Paget’s tooth; lane 3, osteoporosis tooth, with minimal cross-reactivity in the no primary control (composite image of nitrocellulose strips). α-His denotes positive control (anti-His-tag) antibody. Lower molecular weight bands presumably represent a combination of primary antibody reactivity with truncated EBNA-1 protein, and non-specific binding of secondary antibodies to antigen contaminants.

To determine if purified ancient human antibodies preserved any evidence of immune reactivity, as proof-of-concept we tested for proxy function by western blotting against Epstein-Barr virus (EBV) antigen, a ubiquitous human herpesvirus which today infects almost all humans worldwide. After infection, circulating IgG specific for antigens such as EBV nuclear antigen-1 (EBNA-1) remain present for the life of the host and indicate past infection. Although clinically challenging for distinguishing acute from past contemporary infection, this offers opportunities for the identification of ancient latent conditions. To determine if the ancient antibodies retained antigen-recognition ability, western blots were conducted using purified ancient antibodies as primaries, against purified recombinant His-EBNA-1 protein as antigen. Clear cross-reactivity was seen with the antibodies from the putative osteoporosis and RA-teeth, with minimal reactivity in the antibodies from the putative Paget’s tooth or no primary control (Figure 2). Overall, we confirm that ancient antibodies are not only intact but can apparently retain functionality, presumably here representative of ancient systemic host inflammatory responses.

## Discussion

Here, we demonstrate the recovery of whole, intact, disulphide-linked antibody molecules from medieval human teeth, and in exploratory analyses extend to much older samples of mammoth femur bone. Protein sequence data supports the former antibodies as being of human origin, with immunoreactivity on western blot against contemporary EBV protein antigen (EBNA-1) indicating preservation of at least a degree of immune function in centuries old purified antibodies.

Not only can ancient antibodies recognize modern antigens (this work), but conversely immunohistochemistry analyses previously revealed that Mesozoic fossil tissues react with modern antibodies, further evidencing protein preservation in the fossil record. In earlier work, immunofluorescence was used to evaluate *T. rex* bone collagen protein sequences.^10^ Similarly, Eocene deposits have revealed sea turtle fossil bone keratins.^11^ These and other precedents^12^ contextualize our present approach to the identification of ancient antibodies.

Calcified tissues within archaeological remains are rich in mineral components, like hydroxyapatite, which create a high preservation environment, binding antibodies, and other proteins with high affinity. Given that teeth (and indeed bones) are highly vascularized, they may preserve blood and its serum phase, and are potential ancient reservoirs of blood-borne pathogens and host immune responses. Previous paleoproteomic profiling of ancient dental pulp has detected various blood proteins, with imaging analyses indicating that ∼2000-yo pulp samples can preserve intact blood cells from the time of death.^13^ The most likely immunoglobulin to be detected is the most abundant antibody class, IgG, which mediates adaptive immune responses. Indeed, immunoglobulin alpha and gamma sequences of human origin were previously identified by LC-MS/MS from 300-yo human dental pulp isolated from skeletons in France, although the study focused on *Yersinia pestis* proteins also within the samples.^14^ In a study on relatively recent archaeological bone (∼200-yo), ancient IgG was extracted with apparent preserved immunoreactivity against syphilis antigens, as assessed by simple ELISA.^15^ Other reports document detection of intact ancient IgG heavy chains from older ancient bone (e.g., Neolithic) by reducing SDS PAGE and western blotting^16^ and suggest chronic scurvy in juveniles may be associated with reduced IgG recovery. A much older IgG was immunologically detected in samples of 1.4–1.6 million-yo human humeri via dot-blotting.^17^ That report was notable for the exceptional age of the samples, but no attempt at purifying or determining functional reactivity of the IgG was made. “Paleoserology” analyses, making use of a “mini-line blot assay” that involves viral antigens on nitrocellulose incubated with “paleosera” containing ancient protein extracts from human dental pulp, found evidence of a specific immunological response against modern-day Covid sequences, consistent with proteomic evidence of viral sequences.^18^ Notably, the previous proteomic analysis which detected over 100 different proteins from a 43,000-yo mammoth femur^4^ identified three paralogous elephant immunoglobulin heavy chain C proteins orthologous to human IGHG1, a protein detected with over 50% sequence coverage in our study (Figures 1D; Table 2).

However, as noted earlier, the most recent detailed analysis, using different protein extraction methods in parallel, concluded poor ancient antibody recovery, evidence of protein degradation, and overall unsuitability of ancient human IgG proteins from bone, as targets for detecting historical antigens.^7^ That work applied thiophilic adsorption chromatography (TAC), a “gentle” affinity resin method for purification of IgG, that avoids harsh (acidic) elution conditions for purified antibodies. In contrast, by applying a protein G affinity purification step to protein extracts from decalcified medieval human teeth, in our study we find that intact whole antibodies can be routinely recovered, with retention of disulphide linkages indicated from comparisons of western blots performed under reducing versus non-reducing gel electrophoresis. This indicates that the preservation of endogenous protein post-translational modification of ancient proteins extends from phosphorylation^3^ to disulphide bridges. We purified such antibodies on independent occasions from teeth taken from different skeletons with different putative pathologies. Although antibody recovery varied between samples, we have no evidence that this represents anything beyond differential preservation of samples, rather than effects associated with putative pathologies. EBV antigen reactivity of purified antibodies by western blotting was also variable, being strongest in the antibodies purified from the tooth from the skeleton showing signs of osteoporosis. We attribute minimal reactivity against EBNA-1 observed in the no primary control (Figure 2) to either low-level non-specific binding, or the fact that EBV can infect non-human species^19^ and so secondary antibodies themselves may harbor low-level reactivity. Regardless, this is suggestive that at least a degree of functional activity (antigen recognition) can be preserved in ancient antibodies.

The concept of ancient antibodies preserving a molecular memory of an individual’s immune status at the time of death that can potentially be “read” with contemporary immunological approaches is certainly very attractive. This would provide new insights into the long-term association between host immune factors and ancient environmental microbes or changing environments and more broadly the natural history of human health and immunity, including autoimmunity. Although the putative pathologies of the skeletal remains from which the teeth were collected are largely irrelevant in the context of EBV antigen reactivity, our initial analyses failed to detect characteristic disease-associated features of the antibodies purified from the putative RA-tooth, namely anti-citrullinated protein-binding activity or IgG-IgG dimers on non-reducing blots, although more sensitive approaches may be required to reveal such features. However, the skeleton may have had a different form of inflammatory arthritis, given the range of contemporary conditions e.g., seropositive rheumatoid, seronegative rheumatoid, and psoriatic arthritis, which might indeed be retrodiagnosed through more extensive analyses. It remains to extend this analysis to other cases and also to reactivity of ancient antibodies against other viral and bacterial antigens representative of infections that were common in medieval times. For example, environmental factors, potentially including unknown viruses, may account for the unusual features we previously reported in some medieval Paget’s disease cases^6^ as well as the decline in occurrence of the disorder in recent years. Functional analyses of ancient antibodies against viral antigens offer the potential to study past exposure to candidate viral pathogens in Paget’s disease, into deep time.

This work also takes important steps toward addressing the similarities between ancient and contemporary antibodies. Diversity of antibody binding sites is generated by a complex combination of combinatorial and junctional diversity with somatic mutation of the rearranged sequences. In particular, the complementarity determining region 3 (CDR3) of the heavy chain shows extensive variability and plays crucial importance in determining antigen specificity.^20^ Therefore, analysis of ancient antibody protein sequences, and comparison with modern antibodies of known specificity, could in the future indicate information about antigen recognition by the ancient forms. For example, an ancient antibody with high homology to CDR3 of a contemporary antibody that also binds EBNA-1 would be entirely rational. While much of this work remains to be done and requires application of “error tolerant” proteomic approaches that do not rely on matching contemporary sequences in protein databases, the data presented here indicate that human antibodies and their corresponding antigens have both been conserved throughout history, despite the rapid co-evolution of host-pathogen interaction molecules. In situations where ancient pathogens cannot be recovered, ancient antibodies may speak to the history of infectious disease, in particular in the pre-antibiotic age.

### Limitations of the study

The study analyzed a relatively small sample of human teeth, reflecting the fact that destructive analyses are required of important archaeological collections and samples. Population-level analyses including of remains with confirmed ancient infections would be a next step. Antibodies were purified from whole teeth—purifications from enamel, dental pulp, and calculus separately would be an important extension study. Proteomic analysis was performed only on the purified polyclonal antibodies from the putative RA-tooth, as proof of concept. The mammoth femur work was exploratory and again merits extended analysis in future studies.

## STAR★Methods

### Key resources table

**Table:** 

REAGENT or RESOURCE	SOURCE	IDENTIFIER
**Antibodies**		

Peroxidase goat anti-human IgG	Sigma	Cat#A6029; RRID:AB_258272
Mouse anti-Histidine tag	BioRad	Cat#MCA1396
Peroxidase rabbit anti-mouse	DAKO	Cat#P0260

**Biological samples**		

Medieval human teeth	Chester Greyfriars excavation at Linenhall (consumed as part of this study)	N/A

**Chemicals, peptides and recombinant proteins**		

Protein G columns (1mL)	Generon	Cat# 42299.01
Protein A columns (1mL)	Generon	Cat#42295.01
Mammalian protease inhibitor cocktail	Sigma	Cat#P8340
LDS loading dye	Invitrogen	Cat#NP0007
4-12% Bis-Tris PAGE gels	Invitrogen	Cat#NP0322
His-EBNA-1 protein	Abcam	Cat#ab138345
ECL Prime	Amersham Biosciences	Cat#RPN2232
ECL Select	Amersham Biosciences	Cat#RPN2235

**Deposited data**		

Scaffold file (SF3) for viewing of LC-MS/MS data from proteomic analysis of SDS PAGE gel slices corresponding to heavy (A1) and light chain (A2) fractions, of purified ancient antibodies, from medieval human putative rheumatoid arthritis tooth	This paper; Mendeley Data	Mendeley Data: https://doi.org/10.17632/d3vy6nmvhp.1
Dataset S1-.raw file containing LC-MS/MS data from proteomic analysis of gel slice corresponding to heavy chain fraction of purified ancient antibodies from putative RA-tooth	This paper; Mendeley Data	Mendeley Data: https://doi.org/10.17632/d3vy6nmvhp.1
Dataset S2-.raw file containing LC-MS/MS data from proteomic analysis of gel slice corresponding to light chain fraction of purified ancient antibodies from putative RA-tooth	This paper; Mendeley Data	Mendeley Data: https://doi.org/10.17632/d3vy6nmvhp.1

### Resource availability

#### Lead contact

Further information and requests for resources and reagents should be directed to and will be fulfilled by the lead contact, Rob Layfield (robert.layfield@nottingham.ac.uk).

#### Materials availability

This study did not generate new unique reagents.

### Experimental model and study participant details

#### Human skeletal remains

As a source of material for ancient protein extraction and antibody purification, three medieval human teeth (C-14 radiocarbon dating from molars as indicated in Table 1) were collected from human skeletons from the Chester Greyfriars 2016 excavation at Linenhall.^8^ Sample size estimations were not relevant. Teeth were selected based on visual interpretation of different putative pathologies of the skeletal remains, and included consideration of conditions where immune dysfunction or alteration might be anticipated. Teeth were not subject to previous procedures beyond visual inspection.

##### Skeleton LP2045-002

Female, 45–49 years, 149.85 ± 3.57 cm. Coffin burial with heavy erosion (including flaking) and some fragmentation, approximately 50–75% complete.

###### Pathology

Rheumatoid arthritis with accompanying osteoporosis, TMD (Temperomandibular Disorder) and healing *cribra orbitalia.*

##### Skeleton LP2045-004

Male, 30–34 years, 172.08 ± 3.27 cm. Shrouded burial with slight surface erosion and minimal fragmentation, >75% complete.

###### Pathology

Schmorl’s nodes, cranial thickening and clavicular changes indicative of Paget’s disease, dental attrition and an abscess. Occupation/activity traits: Bowman, unfused acromion process (right side), pronounced costal syndemoses and insertions of *teres major* and *pectoralis major.*

##### Skeleton LP2045-018

Female, 50–59 years, 150.09 ± 7.16 cm. Shrouded flexed burial with slight surface erosion and extensive fragmentation, >75% complete.

###### Pathology

Osteoporosis, osteoarthritis to cervical facets, and right patella with accompanying eburnation.

### Method details

#### Ancient protein extraction

Ancient proteins were extracted from samples of whole teeth i.e., enamel, pulp and dentine combined, selected on the basis of very low levels of visible calculus, essentially according to Schmidt-Schultz & Schultz^16^ with the omission of the final TCA precipitation stage. Powdering of teeth was achieved under permanent cooling in liquid nitrogen using a pestle and mortar. Mineralized tooth powder was extracted with buffer A (4 M guanidine-HCl, 20 mM NaH_2_PO_4_, 30 mM Na_2_HPO_4_, pH 7.4; 250 μL per 50 mg powder) with addition of a mammalian protease inhibitor cocktail (Sigma, P8340, 1:1000) under permanent mixing for 1 day (4°C). After centrifugation (10,000g, 30 min, 4°C), the supernatant was discarded. The pellet was further extracted with 250 μL (per 50 mg original powder) buffer B (buffer A and 300 mM EDTA with inhibitors) under constant stirring for 1 day (4°C). After centrifugation (10,000g, 30 min, 4°C), chelated calcium ions were removed from the resulting pellet by washing three times with autoclaved double-distilled water and centrifuging (10,000g, 20 min, 4°C). The washed pellet was sonicated on ice (3 × 10s) in a neutral 50 mM phosphate buffer pH7.4 (250 μL per 50 mg original powder) and 10 mM EDTA, with extracted proteins being solubilized by the phosphate buffer. After a final centrifugation (35,000g, 20 min, 4°C) phosphate-extracted proteins (in the supernatant) were retained. All procedures were performed with gloves, autoclaved instruments, and autoclaved or sterile filtered solutions. Blinding was not relevant as this was not a comparative analysis.

#### Affinity purification of ancient antibodies

Purification of human antibodies was via protein G whilst mammoth purifications were via protein A. Briefly, for protein G purifications, a 1 mL column (Generon) was used with protein samples diluted 1:1 (to a total volume of 500 μL) with binding buffer (100 mM sodium phosphate, 150 mM NaCl, pH 7.2). Columns were washed initially with 10 mL of binding buffer before diluted samples were injected via the loop function of the AKTA Start FPLC system (GE Healthcare) to the column at 1 mL/min. Flow through was collected and the column washed with a further 6 column volumes of binding buffer to remove unbound proteins. Samples were eluted using 5 column volumes of 0.1 M glycine pH 2.3 and neutralized using 500 μL of 1M Tris (pH 8). Fractions were pooled, concentrated, and dialyzed against PBS. Protein A purification was as above with binding buffer replaced with 20 mM sodium phosphate pH 7.2 and elution buffer replaced with 0.1M citric acid pH 3.0. Blank antibody purifications were performed in parallel, with the substitution of teeth protein samples with binding buffer. Failure to detect signals in blank purifications was interpreted as the absence of any contamination of the experimental archaeological materials.

#### SDS PAGE and western blotting

For SDS PAGE analysis of tooth total protein extracts, proteins samples (phosphate extractions and phosphate-extracted insoluble pellets) were mixed with reducing SDS PAGE loading buffer (1:1 for phosphate extractions), heated at 95°C for 5 min, then loaded onto a gradient SDS PAGE gel (Tris-glycine, 5–20% acrylamide; 70 μL per lane). Samples were electrophoresed and total proteins detected by silver stain.

For Western blot detection of purified antibodies as antigen, samples for reducing gel electrophoresis were prepared by the incubation of purified antibodies with 0.05 M TCEP for 15 min at 95°C, diluted 1:4 with loading dye (LDS, Invitrogen). Non-reducing samples were prepared diluted 1:4 with LDS but without heating or reducing agent. Samples (∼5 μg) were applied to a 4–12% Bis-Tris PAGE Gel (NOVEX, Invitrogen) and electrophoresed at 165V in MES buffer (Invitrogen) using the BOLT system for 30 min. Transfer after SDS PAGE was carried out using the BOLT system, gels were first washed in dH_2_O and then transfer buffer (Invitrogen, 15% (v/v) methanol). PVDF membranes were activated in methanol (2 min, room temperature) and transfer carried out in the BOLT transfer cassette at 10V for 1 h. Membranes were washed in PBS-Tween 20 (0.1% (v/v)) and blocked with semi-skimmed milk (5% (w/v) in PBS-Tween) for 1 h agitating at room temperature. Membranes were probed with peroxidase-conjugated goat anti-human IgG (Sigma, A6029) at a dilution of 1:5000 in 5% (w/v) semi-skimmed milk overnight at 4°C. Exposure was using ECL Prime or Select as appropriate (Amersham Biosciences). Sequential exposures were carried out to determine optimal timepoints. Purified antibodies were analyzed by western blotting on at least two independent occasions. Sufficient cross-reactivity was seen with A6029 to also allow the use of this antibody for purified mammoth antibody detection under the same conditions.

For western blotting with purified antibodies as primaries, purified recombinant His-EBNA-1 protein (Abcam, ab138345) was used as antigen. 2 μg aliquots of protein antigen were separated by SDS PAGE (Tris-glycine, 5–20% acrylamide) and transferred to nitrocellulose membranes. Membranes were blocked with 5% (w/v) semi-skimmed milk in TBS-Tween (0.05% (v/v)) and probed with 50 μL purified ancient protein in 500 μL total volume of blocking solution (i.e., 1:10 dilution). The negative control (no primary) was incubated with blocking solution only. Membranes were probed with 1:1000 dilution of secondary peroxidase-conjugated goat anti-human IgG (Sigma, A6029). Positive control was probed with 1:10000 mouse anti-Histidine tag antibody (Bio-Rad, MCA1396) and secondary rabbit anti-mouse (DAKO, Agilent, P0260) diluted 1:5000. Western blot analyses were repeated on multiple independent occasions (n = 3).

#### Sample preparation for LC-MS/MS analysis

For preliminary tooth whole proteome analyses, protein samples (phosphate extractions) as above were mixed with reducing SDS PAGE loading buffer and after heating, loaded onto a gradient SDS PAGE gel (Tris-glycine, 5–20%; 70 μL per lane). Gels were electrophoresed at 600V, 45mA for 10–15 min, to run the complete mixture of extracted proteins into the top few millimeters of the resolving gel. Gels were stained with Coomassie blue overnight and destained using 10% (v/v) methanol, 10% (v/v) acetic acid. Slices were cut from the gel (containing the complete complement of solubilized proteins) using a clean scalpel for each sample and placed into a clean labeled Eppendorf with 100 μL deionized water. For targeted proteomic analysis of purified ancient antibodies (from the putative RA-tooth), a similar procedure was followed, although gels were fully electrophoresed until the dye front reached the bottom of the resolving gel, to separate antibody heavy and light chain (∼5 μg total protein). Following Coomassie staining, antibody heavy (∼60 kDa) and light chains (∼25 kDa) were separately excised in polyacrylamide gel slices, as for total proteome analyses.

#### Generation and analysis of proteomic data

LC-MS/MS analysis (Cambridge Center for Proteomics) was used to catalog purified proteins within polyacrylamide gel slices, using tryptic digestion. Coomassie-stained protein bands from SDS PAGE were excised, reduced, alkylated, digested with trypsin and submitted to MS/MS on an LTQ Orbitrap spectrometer with nanoflow liquid chromatography (LC). Peptides were identified in data-dependent mode, using Mascot (2.7.0) to search the human UniProt (2018) and cRAP (2019) databases (93734 proteins, 2 max missed cleavage sites, precursor ion mass tolerance 20 PPM, fragment ion mass tolerance 0.100 Da) and validated with Scaffold (4.11.1). Carbamidomethyl (+57 on C) was considered a fixed modification, while deamidation (+1 on N/Q), oxidation (+16 on M), phosphorylation (+80 on S/T/Y), and acetylation (+42 on K/N-terminal M) were considered as variable modifications. Proteomic data was visualized using Scaffold 4. In total 206 proteins were identified in 152 clusters across the two samples (heavy and light chain fractions from the putative RA-tooth) of purified ancient antibody (9.2% FDR; protein threshold 99.0%, minimum number of peptides = 2, peptide threshold 95%). Blank runs were performed before and between LC runs.

### Quantification and statistical analysis

Statistical analysis is an integral feature of the Scaffold (proteomic data viewer) software used. Briefly, Scaffold uses Bayesian statistics to estimate the peptide probabilities, and calculates protein probabilities by combining the probabilities of all the peptides in the protein. For a detailed description see: https://support.proteomesoftware.com/hc/en-us/articles/115002213066-Scaffold-and-Assumptions-Regarding-Statistical-Analysis-.

## Data Availability

• Proteomic data has been deposited at Mendeley Data and is publicly available as of the date of publication. Accession numbers are listed in the key resources table. • This paper does not report original code. • Any additional information required to reanalyze the data reported in this paper is available from the lead contact upon request.
