# Supplementary material for: Preservation of whole antibodies within ancient teeth
Source: iScience. 2023 Aug 9;26(9):107575. doi: 10.1016/j.isci.2023.107575 (PMC10445445; doi:10.1016/j.isci.2023.107575)
Supplement: Document S1. Figures S1 and S2 [file mmc1.pdf]

iScience, Volume *26*

Supplemental information

Preservation of whole antibodies

within ancient teeth

Barry Shaw, Thomas McDonnell, Elizabeth Radley, Brian Thomas, Lynn Smith, Carol A.L. Davenport, Silvia Gonzalez, Anisur Rahman, and Rob Layfield

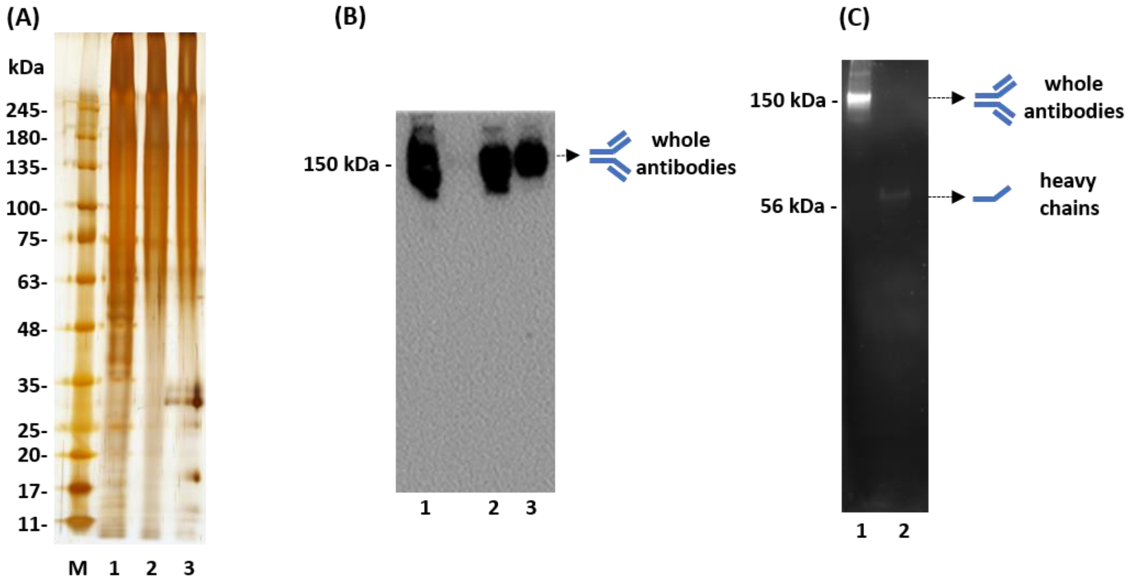

Figure S1: Purification of whole ancient antibodies from human Paget's and osteoporosis teeth, related to Figure 1. A.) Representative SDS PAGE analysis (silver stain of phosphate extracts) of proteins extracted from medieval human teeth (M, molecular weight marker; lane 1, putative RA-tooth; 2, putative Paget's tooth; 3, putative osteoporosis tooth). B.) Western blot under non-reducing conditions of affinity purified antibodies from: lane 1, control modern human serum; 2, putative osteoporosis tooth; 3, putative Paget's tooth) with antibody structure noted. C.) Western blot under non-reducing (lane 1) or reducing (lane 2) conditions of affinity purified ancient antibodies from putative Paget's tooth, with different antibody structures noted.

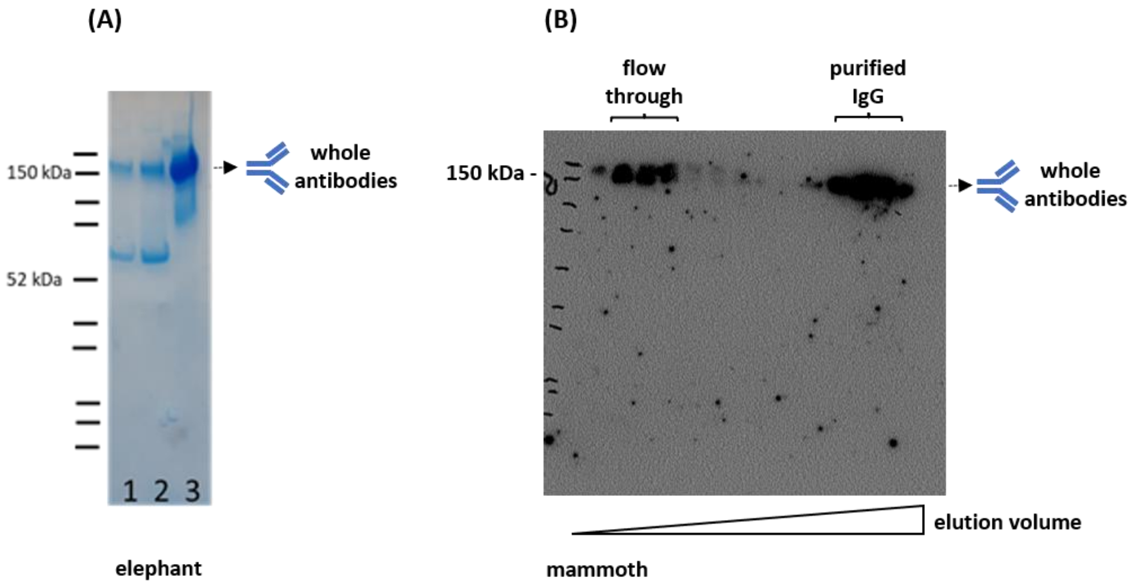

Figure S2: Purification of whole ancient antibodies from mammoth bone, related to Figure 1. A.) SDS PAGE analysis under non-reducing conditions showing protein A (lane 3) is superior to protein G (lanes 1, 2) for the affinity purification of control elephant IgG from serum. B.) Fractions of protein A-purified antibodies from a sample of mammoth femur, western blotted under non-reducing conditions, show evidence of whole intact antibodies across different fractions from an FPLC elution (purified IgG), with unbound antibody (flow through) also indicated. Western blot detected with peroxidase-conjugated goat anti-human IgG, which shows cross-reactivity with mammoth IgG.
